# Advanced Biliary Tract Cancer: Exploration of Third-Line and Other Therapeutic Areas After Failure of Chemotherapy Alone

**DOI:** 10.3390/cancers17193268

**Published:** 2025-10-09

**Authors:** Li Ma, Cheng Yi

**Affiliations:** Division of Abdominal Tumor, Department of Medical Oncology, Cancer Center, West China Hospital, Sichuan University, Chengdu 610041, China; mali24@stu.scu.edu.cn

**Keywords:** biliary tract cancer, third-line therapy, tumor markers, FOLFOX, microorganisms

## Abstract

**Simple Summary:**

The diagnosis and treatment of advanced biliary tract cancer face numerous challenges, including the lack of highly specific diagnostic methods, insufficient healthcare system support, and the absence of standardized and effective treatment options after the failure of multiple lines of therapy. This review aims to improve the prognosis of biliary tract cancer by presenting standard treatment regimens, clinical trials, and experimental findings, thereby providing new insights for subsequent-line therapy in advanced disease.

**Abstract:**

Biliary tract cancer (BTC) is a highly aggressive malignancy with an extremely poor prognosis and a gradually increasing incidence, warranting increased clinical attention. The majority of BTC patients are diagnosed at an unresectable stage, making systemic therapy—including first-line and subsequent treatments—critical for outcomes. However, due to disparities in medical resources and limited understanding of the disease, outcomes following first- and second-line therapies remain suboptimal. In this context, third-line treatment offers a potential opportunity to further extend patient survival, although challenges such as poor treatment tolerance and significant drug-related toxicities remain. A rational integration of chemotherapy, targeted therapy, immunotherapy, and novel radiotherapy techniques may constitute a standardized third-line therapeutic strategy for BTC. This review aims to discuss potential therapeutic adaptations and options in the setting where conventional chemotherapy has failed.

## 1. Introduction

Biliary tract carcinoma (BTC) is a highly aggressive and heterogeneous malignant tumor originating from the biliary epithelium. It is anatomically classified into gallbladder carcinoma (GBC), intrahepatic cholangiocarcinoma (iCCA), perihilar cholangiocarcinoma (pCCA), and distal cholangiocarcinoma (dCCA). Among these, pCCA and dCCA are often collectively referred to as extrahepatic cholangiocarcinoma (eCCA). As the second most common primary malignant liver tumor, BTC has been exhibiting a steadily increasing incidence worldwide, leading to a growing disease burden.

The epidemiological distribution of BTC demonstrates significant geographic and demographic variations. Countries such as Chile, Japan, and Korea report a high prevalence of BTC. Recent epidemiological data further reveal that East Asia [1], particularly northeastern Thailand, has the highest age-standardized incidence rate (ASR) of iCCA globally, reaching 85 per 100,000 population. The widespread endemicity of liver fluke infection in this region, despite available anthelmintic treatments, remains a major risk factor for iCCA due to its persistent long-term carcinogenic potential. In contrast, GBC imposes the heaviest disease burden in Chile, with an age-standardized incidence rate (ASIR) as high as 10.38 per 100,000. Although the incidence in East Asia [2] (ASIR ≈ 3.0 per 100,000) is lower than that in Chile, the mortality rate (age-standardized mortality rate [ASMR] = 2.4 per 100,000) remains relatively high, reflecting its poor prognosis and disparities in diagnosis and treatment. Additionally, high body mass index (BMI), high-income populations in North America, and individuals aged ≥50 years have been identified as high-risk groups for BTC [3].

**Figure 1:**
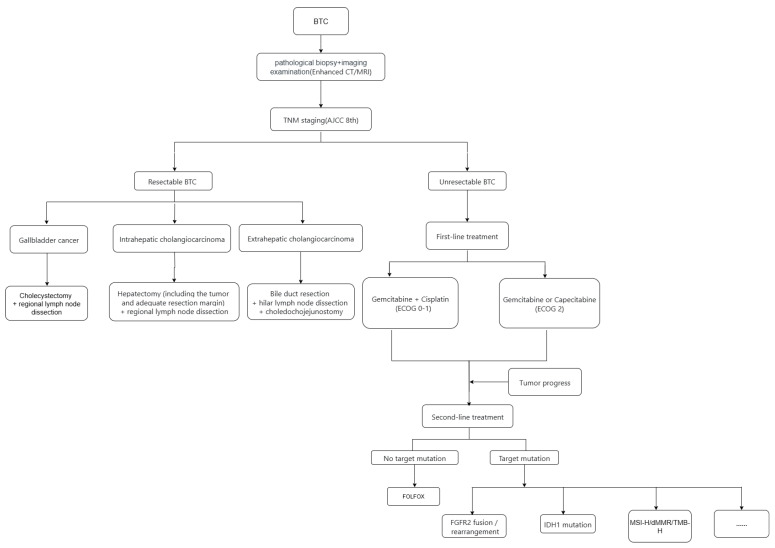
Treatment Algorithm for Biliary Tract Cancer (BTC).

Although the overall incidence of BTC has been rising over the past few decades, one study [4] indicates that the risk of developing and dying from the disease remains relatively low among young adults aged 15–39 years. Nonetheless, the prognosis of BTC is generally poor, with over 70% of patients diagnosed at an advanced stage, precluding curative surgical intervention. Currently, gemcitabine plus cisplatin-based first-line therapy (Figure 1) and second-line regimens such as FOLFOX have achieved certain consensus in clinical practice. However, once disease progression continues, there remains a lack of standardized and effective third-line treatment strategies, posing a significant challenge in clinical management (Figure 1).

In this context, this article provides a systematic review of the latest strategies and research advances in third-line treatment for advanced BTC. By combining keywords such as “advanced biliary tract cancer”, “metastatic cholangiocarcinoma”, “gallbladder cancer”, “cholangiocarcinoma”, “immunotherapy”, “targeted therapy”, and “clinical trials”, we summarize relevant clinical studies from the past five years. The aim is to offer evidence-based guidance for individualized treatment selection in later-line settings and to outline future research directions.

## 2. Tumor Biomarkers

The latest EASL-ILCA Clinical Practice Guidelines clearly delineate the comprehensive pathway for iCCA management, spanning from diagnosis to treatment. However, the outlined diagnostic approaches remain heavily reliant on macroscopic tumor identification or well-established clinical histories, as evidenced by recommended procedures such as tumor biopsy and molecular profiling for high-risk recurrence populations noted in the guidelines. This limitation is similarly reflected in the ESMO guidelines [5].

There is a current need for more efficient biomarkers for disease diagnosis. Currently, the early diagnosis of biliary tract carcinoma (BTC) remains a significant challenge. Approximately half of the patients lack typical clinical symptoms, and while commonly used serum markers such as CA19-9 are elevated in about 70% of advanced cases, their sensitivity and specificity are limited due to frequent abnormalities in benign biliary diseases, restricting their diagnostic value. The 2025 updated ESMO guidelines further highlight the current absence of BTC-specific HER2 assessment criteria [5]. Therefore, the development of novel biomarkers with high sensitivity and specificity is of great importance for improving early detection and dynamic monitoring of BTC.

Among emerging diagnostic markers, cytokeratin 19 fragment (CYFRA 21-1) demonstrates considerable promise. Studies indicate that its specificity surpasses that of CA19-9 (at a cutoff ≥1.5 ng/mL: 88% vs. 78%; ≥3.0 ng/mL: 97% vs. 95%) [6]. Combined detection of both markers further increases specificity to 96%, indicating good complementarity. Although carcinoembryonic antigen (CEA) is commonly associated with colorectal cancer, it is also elevated in approximately 30% of cholangiocarcinoma patients [7], suggesting its potential value as an auxiliary marker. Epigenetic markers such as Hypermethylation of the CpG island associated with the HOXD8 gene have demonstrated 100% sensitivity and specificity in both tissue and bile samples, showing potential as an ideal non-invasive diagnostic tool [8].

Some biomarkers also exhibit value in predicting treatment response. For instance, a study [9] conducted at West China Hospital in China showed that patients with DDR-positive status (defined as the presence of ≥1 deleterious mutation, such as frameshift, nonsense, or splice-site mutations) exhibited greater sensitivity to platinum-based chemotherapy and combined immunotherapy, suggesting its potential as a predictive biomarker for treatment efficacy, though further validation through large-scale prospective studies is still required. Within liquid biopsy techniques, circulating tumor DNA (ctDNA) enables non-invasive dynamic monitoring. Kearney J.F. et al. [10] noted that bile-derived ctDNA has a higher mutation detection rate than plasma-derived ctDNA, making it more suitable for mutational profiling and guiding individualized medication.

Accurate prognosis assessment is equally critical for optimizing the clinical management of BTC. Elevated osteopontin levels are associated with shorter patient survival, a mechanism potentially linked to the activation of the Wnt/β-Catenin signaling pathway and the promotion of metastasis; intervention in this pathway can inhibit these effects [11]. MUC5AC, a mucin protein, shows that elevated serum levels are correlated with poor prognosis in intrahepatic cholangiocarcinoma (iCCA), though the significance of its tissue expression remains controversial, and it currently cannot be used to differentiate BTC from other gastrointestinal tumors [12].

M6A RNA methylation modification, dynamically regulated by factors such as METTL3 (writer), FTO (eraser), and YTHDF2 (reader), plays a key role in RNA metabolism and is crucially involved in the pathogenesis and progression of BTC. Bai X. et al. [13] indicated that abnormal expression of m6A-related genes could serve as diagnostic and prognostic markers; Huang C. et al. [14], based on a 520-gene panel sequencing and multi-lineage cytokine analysis, identified TP53, NRAS, FBXW7, and APC as key prognosis-related genes. In advanced patients treated with PD-1 inhibitors, sequence entropy and mutational signatures demonstrated superior prognostic stratification ability compared to tumor mutational burden (TMB). Furthermore, enrichment of TP53/KRAS/NRAS mutations and high CXCL10 expression in plasmacytoid dendritic cells (pDCs) are also associated with poor prognosis.

Clinicopathological factors also carry significant prognostic implications. Rohan T. et al. found that tumor size > 5 cm, distant metastasis, high bilirubin, and high CRP levels were poor prognostic factors in cholangiocarcinoma patients undergoing percutaneous transhepatic biliary drainage (PTBD) [15]. A population-based study [16] in South Korea showed that ampullary carcinoma had the best 5-year relative survival rate (48.5%) due to its high proportion of localized cases (78.2%), while intrahepatic cholangiocarcinoma (iCCA) patients had the lowest rate of receiving active treatment (52.8%) and the worst survival (10.8%). Compared to younger patients, elderly patients are more inclined to refuse active treatment, suggesting that enhanced management of treatment intentions in older patients may help improve their prognosis. Mass spectrometry techniques, such as MALDI-TOF MS for detecting peptide mass fingerprints (PMFs) and LC–MS/MS for peptide biomarker identification, can improve the accuracy of recurrence classification in iCCA and hold potential for guiding individualized recurrence therapy [17].

## 3. Limitations

The biliary system is anatomically deeply situated and adjacent to critical vascular structures and surrounding organs, making surgical resection technically challenging and high-risk. Only approximately 20% of patients are eligible for curative resection at initial diagnosis [18], and there is currently no internationally unified standard for surgical strategies [19]. A study [20] reflecting the current state of biliary tract cancer diagnosis and treatment in Europe indicated that even after undergoing curative surgery, the 5-year overall survival rate remains only between 20% and 35%. This multicenter study further emphasized that a multidisciplinary team (MDT) approach significantly improves the quality of clinical decision-making and patient outcomes, representing a key strategy in addressing the complexity of BTC.

Clinical practices for BTC vary significantly across different global regions, primarily due to disparities in economic development, allocation of medical resources, drug approval policies, and cultural contexts. Compared to the U.S. FDA, the drug review process by the European Medicines Agency (EMA) is generally more cautious and protracted, leading to delays in the availability of novel therapies in Europe. Moreover, aside from a few countries such as Germany, many European regions face significant delays in the reimbursement of anticancer drugs, substantially impacting treatment accessibility. Central and Eastern Europe lag in medical infrastructure, molecular testing capabilities, and specialized training, further exacerbating regional disparities in diagnostic accuracy and equity of healthcare delivery. Currently, about 25% of medical institutions across Europe still cannot perform molecular pathological testing independently and must rely on external laboratories, which may delay treatment and hinder the implementation of personalized medicine.

To address these challenges, Europe is actively promoting several regional and national healthcare optimization initiatives [21]. Efforts to standardize clinical pathways through pan-European collaboration mechanisms, along with policy coordination to accelerate new drug approvals and insurance coverage integration, have gradually emerged as important strategies for improving the diagnostic and treatment system. Simultaneously, an international project named “BABEL” [22] is being implemented with the goal of developing standardized educational and patient guidance materials for biliary tract cancer chemotherapy. These materials will be available in 208 languages and dialects, specifically targeting low- and middle-income countries and patient populations with language barriers. This initiative aims to eliminate health information inequality caused by language barriers, enhance the standardization and accessibility of BTC diagnosis and treatment worldwide, and represents an innovative effort with significant public health implications.

## 4. First-Line and Second-Line Treatment

The first-line treatment of biliary tract cancer (BTC) continues to face challenges due to progressively declining disease control rates, primarily due to cumulative toxicity from conventional chemoradiotherapy and enhanced mechanisms of tumor drug resistance. The current standard first-line regimen consists of gemcitabine plus cisplatin (GemCis), the efficacy of which was established in the ABC-02 trial [23]. This study demonstrated that the combination therapy significantly prolonged median overall survival (mOS) compared to gemcitabine alone (11.7 months vs. 8.1 months, *p* < 0.001). The latest EASL-ILCA Clinical Practice Guidelines also endorse this perspective. In recent years, first-line strategies have been continuously optimized to further improve outcomes (Table 1). Although molecular targeted therapies have shown efficacy in specific subgroups (Table 2), such as patients with IDH1 or FGFR2 alterations, their applicability remains limited to narrow populations, lacking broad generalizability. Therefore, the introduction of immune checkpoint inhibitors has become a pivotal strategy to improve outcomes across a broader patient population.

The TOPAZ-1 trial [24] confirmed that adding the PD-L1 inhibitor durvalumab to GemCis further extended mOS to 12.9 months (compared to 11.3 months in the control group) and increased the objective response rate (ORR) from 18.9% to 26.7%. The EASL-ILCA Clinical Practice Guidelines affirm that the addition of durvalumab, where applicable, enhances therapeutic efficacy. Similarly, the KEYNOTE-966 trial [25] demonstrated that the addition of the PD-1 inhibitor pembrolizumab to GemCis resulted in a significant improvement in mOS compared to chemotherapy alone (12.7 months vs. 10.9 months). These two Phase III studies collectively establish immune checkpoint inhibitor combination chemotherapy as the new standard first-line treatment, marking the entry of BTC into a new era of immunotherapy.

The duration of response to first-line therapy is often limited, underscoring the growing importance of second-line treatment. For patients without actionable molecular targets, the FOLFOX regimen (oxaliplatin + leucovorin + 5-fluorouracil) represents the current standard second-line chemotherapy option [26], though its efficacy remains debated. The ABC-06 trial indicated that compared to active symptom control, FOLFOX provided only a modest survival benefit (mOS: 6.2 months vs. 5.3 months; HR = 0.69, *p* = 0.031), and its clinical relevance remains uncertain. Notably, for patients with actionable therapeutic targets, the EASL-ILCA Clinical Practice Guidelines emphasize that those with dMMR/MSI-H may benefit from immune checkpoint inhibitors, while patients harboring FGFR2 fusions/rearrangements could derive clinical benefit from FGFR inhibitors. These favorable outcomes are consistently observed in both first-line and second-line treatment settings. In comparison with the EASL-ILCA guidelines, the ESMO guidelines recommend a broader spectrum of targeted therapies (as referenced in Table 2), including larotrectinib/entrectinib and dabrafenib, which have not yet received FDA or EMA approval for CCA treatment [5]. Beyond FOLFOX, other regimens such as XELIRI (capecitabine + irinotecan) and FOLFIRI are under active investigation. A single-center randomized phase II study [27] conducted in China involving 60 patients who progressed after first-line therapy compared XELIRI with irinotecan monotherapy. The results demonstrated that XELIRI significantly improved median progression-free survival (mPFS: 3.7 months vs. 2.4 months; *p* = 0.036) with a tolerable safety profile. Moreover, more patients in the XELIRI group (43.3%) went on to receive third-line therapy, suggesting that this regimen may provide a broader therapeutic window.

The FOLFIRI regimen represents another notable second-line option as it avoids the peripheral neurotoxicity associated with oxaliplatin. A small-scale Belgian study [28] involving 12 BTC patients with extrahepatic metastases reported an mPFS of 1.7 months (95% CI: 0.66–2.67) and an mOS of 5 months (95% CI: 2.77–7.20) with manageable toxicity and no grade 4 or higher adverse events.

In summary, chemotherapy remains the cornerstone of BTC management. While first-line immunotherapy combined with chemotherapy has significantly improved survival, various second-line chemotherapeutic regimens also demonstrate potential (Table 3). However, given the challenges of disease progression and complex resistance mechanisms, no standardized third-line therapy currently exists. More high-quality clinical studies are needed to explore optimal treatment strategies, personalized approaches, and novel drug combinations to further improve outcomes in patients with advanced BTC. The EASL-ILCA Clinical Practice Guidelines primarily focus on first-line and second-line treatment regimens, with limited coverage of highly innovative or low-yield research—a characteristic likely inherent to its nature as clinical guidelines. In contrast, the ESMO guidelines appear to encompass a broader range of methodologies. Notably, as indicated in the guidelines, certain therapies with low levels of evidence still require further investigation. Clinical guidelines undoubtedly reflect current trends and challenges in the management of biliary tract malignancies. First, they highlight the importance of international collaboration and multicenter initiatives in addressing the issue of small sample sizes in existing studies. Second, they advocate for the development of biomarkers to guide diagnosis, prognosis, and treatment decisions. When synthesizing the content of these guidelines, it becomes evident that current research often prioritizes first-line therapies while paying less attention to the exploration of later-line treatments. Interestingly, patients who qualify for later-line therapies represent a distinct subset of those with biliary tract cancer. Small sample sizes should not justify their exclusion from focused research efforts!

## 5. Identification of Therapeutic Targets

In recent years, significant progress has been made in targeted therapy and immunotherapy for advanced biliary tract cancer (BTC). Multiple targeted agents directed against specific molecular alterations have entered clinical evaluation and demonstrated encouraging efficacy. For instance, drugs such as pemigatinib and futibatinib for FGFR2 fusions/rearrangements, ivosidenib for IDH1 mutations, and trastuzumab for HER2-positive tumors have been evaluated in phase II or III clinical trials and received corresponding regulatory approvals. Unlike traditional chemotherapeutic agents that broadly affect rapidly dividing cells, these targeted therapies specifically recognize and bind to molecular targets on tumor cells, thereby precisely inhibiting pro-proliferative signaling pathways, which enhances efficacy while reducing systemic toxicity.

Key clinical trial results showed that in previously treated patients with FGFR2 alterations, pemigatinib achieved an objective response rate (ORR) of 37%, a median progression-free survival (mPFS) of 6.9 months, and a median overall survival (mOS) of 21.1 months [29]; futibatinib yielded an ORR of 41.7%, mPFS of 8.9 months, and mOS of 20.0 months [30]; whereas ivosidenib treatment in IDH1-mutant patients resulted in an ORR of 2.4%, mPFS of 2.7 months, and mOS of 10.3 months [31]. However, these agents showed limited efficacy in patients without the corresponding molecular alterations, underscoring the importance of precise molecular stratification. Similarly, immune checkpoint inhibitors such as pembrolizumab achieved a response rate (RR) of 53% in the MSI-H/dMMR subgroup, compared to only 3–13% in unselected BTC populations, further highlighting the significant heterogeneity in treatment response and emphasizing the need to explore novel targets and combination strategies to benefit more patients.

Advances in molecular mechanism research have provided critical support for target discovery. Nakamura H. et al. [32] performed multi-omics sequencing on 260 Japanese BTC cases and found that APOBEC-related mutations were more common in gallbladder cancer and extrahepatic cholangiocarcinoma, FGFR2 fusions were predominant in intrahepatic cholangiocarcinoma, and PRKACA/PRKACB fusions were frequently observed in extrahepatic cholangiocarcinoma. Studies in animal models revealed that mutations in genes such as Trp53, Fbxw7, Inppl1, Tgfbr2, and Cul3 promoted the development of intrahepatic cholangiocarcinoma [33]. Among these, Fbxw7 mutations may predict sensitivity to immunotherapy, while Tgfbr2 mutations could enhance response to FGFR inhibitors.

ERBB2 represents one of the most promising targets in gallbladder cancer; however, primary or acquired resistance to trastuzumab is common. A study [34] from China identified a novel splice variant, ERBB2 i14e, which is highly expressed in gallbladder cancer. This variant enhances interaction with ERBB3, activates the AKT pathway, promotes proliferation, and confers trastuzumab resistance through steric hindrance. Inhibition of this splice variant using antisense oligonucleotides restored drug sensitivity. Additionally, the novel fusion gene PUM1-TRAF3 was found to promote tumor progression via activation of the non-canonical NF-κB pathway. High expression of NIK was significantly associated with poor patient prognosis [35] [2-year disease-free survival: 18.0% vs. 62.9% (*p* = 0.001); overall survival: 18.0% vs. 46.3% (*p* = 0.001)], suggesting its potential as both a therapeutic target and prognostic biomarker.

Metabolism-oriented targeted therapy has also shown preliminary progress. Although some epidemiological studies suggested that GLP-1 receptor agonists might increase the risk of cholangiocarcinoma. A preclinical study [36] demonstrated that liraglutide did not promote the proliferation of intrahepatic cholangiocarcinoma cells and significantly reduced metastatic tumor volume (*p* < 0.001) and weight (*p* = 0.046), supporting its continued investigation in diabetic patients with intrahepatic cholangiocarcinoma. Zhen Y. et al. [37] reported that FGFR2 maintains a glycolytic phenotype in tumors through NF-κB activation. Upon FGFR inhibition, tumor cells shifted toward fatty acid oxidation dependency, and combining mitochondrial-targeted agents with intermittent fasting enhanced antitumor efficacy.

Claudin 18.2 (CLDN18.2), a tight junction protein exposed during malignant transformation, was detected in 29.5% of BTC samples in one study [38], with 5.5% showing strong positivity. Heterogeneous expression was particularly observed in extrahepatic cholangiocarcinoma and gallbladder cancer, indicating its potential as an emerging target, though larger studies are needed to validate its clinical value.

The tumor microenvironment has also become a focus of therapeutic research. Lim DH et al. [39] found that activated cancer-associated fibroblasts (aCAFs) were associated with poor prognosis in patients with distal cholangiocarcinoma. Refametinib, a MEK inhibitor, reduced fibroblast activation, suggesting that aCAFs may serve not only as prognostic markers but also as potential therapeutic targets.

In conclusion, targeted and immunotherapy for advanced BTC is still rapidly evolving, with numerous studies contributing to the development of personalized treatment strategies. Future efforts should focus on in-depth mechanistic exploration, innovative combination therapies, and optimization of biomarker selection to improve patient outcomes.

## 6. Clinical Trials in Treatment-Resistant Settings

### 6.1. Chemotherapy

In a multicenter, randomized, open-label phase IIb clinical trial [40] conducted in Korea, investigators evaluated the efficacy and safety of liposomal irinotecan (nal-IRI) combined with 5-fluorouracil/leucovorin (5-FU/LV) compared to 5-FU/LV alone as a second-line treatment in patients with advanced biliary tract cancer (BTC). Nal-IRI represents an optimized formulation of irinotecan. All enrolled participants had experienced disease progression following first-line therapy with gemcitabine plus cisplatin, with females accounting for 42.1% of the cohort. Patients were randomized in a 1:1 ratio, resulting in 88 subjects in the combination group and 86 in the control group. Both groups received intravenous 5-FU 2400 mg/m^2^ and LV 400 mg/m^2^ every two weeks, with the experimental arm additionally receiving nal-IRI 70 mg/m^2^.

The results demonstrated that the nal-IRI combination significantly improved the primary endpoint of median progression-free survival (mPFS: 3.9 months vs. 1.6 months; HR = 0.51, 95% CI 0.37–0.71, *p* < 0.001) and the key secondary endpoint of median overall survival (mOS: 8.6 months vs. 5.3 months; HR = 0.68, 95% CI 0.49–0.94, *p* = 0.02) compared to the control group. The objective response rate (ORR) was also significantly higher in the combination group (19.3% vs. 2.3%, *p* < 0.001), along with a superior disease control rate (DCR: 60.2% vs. 29.1%), confirming meaningful antitumor activity. Therefore, the nal-IRI plus 5-FU/LV regimen represents not only an effective second-line option but also a potential strategy for later-line treatment in selected patients.

On the other hand, treatment selection should take into account patient age, performance status, and comorbidities. For instance, gemcitabine plus S-1 (GS regimen)—where S-1 is an oral fluoropyrimidine derivative containing tegafur, gimeracil, and oteracil potassium—may offer advantages in elderly patients. A post hoc subgroup analysis [41] of the randomized phase III trial JCOG1113, stratified by age (≥75 years vs. <75 years), showed that among older patients, the GS regimen resulted in significantly longer overall survival compared to gemcitabine plus cisplatin (GC) (17.7 months vs. 12.7 months), along with a lower incidence of grade ≥ 3 hematological adverse events, indicating a more favorable tolerability profile. These findings underscore the importance of considering age in treatment tolerance and efficacy, supporting the implementation of individualized therapy strategies. Optimizing chemotherapeutic choices in older adults holds significant clinical relevance.

### 6.2. Targeted Therapy

Approximately 5% of biliary tract cancer (BTC) patients harbor BRAF mutations, with V600E being the most common pathogenic variant, which has emerged as an important therapeutic target. The combination of dabrafenib and trametinib has demonstrated significant efficacy across multiple solid tumors carrying the BRAF V600E mutation. A multicenter, single-arm phase II study [42] (NCT02034110) specifically evaluated this combination in 43 previously treated patients with advanced, BRAF V600E-mutant BTC. Patients received dabrafenib 150 mg orally twice daily and trametinib 2 mg once daily until disease progression or unacceptable toxicity. The cohort comprised 56% females, 91% with intrahepatic cholangiocarcinoma, and 93% with stage IVB disease. Results showed a median overall survival (mOS) of 13.5 months (95% CI: 10–33) and a median progression-free survival (mPFS) of 8.9 months (95% CI: 5–10), confirming meaningful clinical benefit in this population. The incidence of grade ≥3 treatment-related adverse events was 21%, with elevated gamma-glutamyltransferase being the most common (12%). Based on these encouraging results, Subbiah V. et al. recommend routine BRAF V600E mutation testing in all advanced BTC patients to enable precision therapy.

However, not all targeted interventions against signaling pathways have proven successful. For example, a randomized phase II trial [43] investigating the addition of the MEK inhibitor selumetinib (administered either continuously or sequentially) to gemcitabine and cisplatin in advanced BTC showed no significant improvement in tumor shrinkage, mPFS, or mOS compared to chemotherapy alone. Moreover, the combination was associated with a higher incidence of grade ≥3 adverse events, suggesting that this strategy is not recommended based on current evidence.

HER2 alterations are found in approximately 5–19% of BTC cases, particularly in gallbladder cancer, providing a rationale for targeted therapy. Zanidatamab, a novel bispecific antibody targeting both extracellular domains ECD4 and ECD2 of HER2, enables dual signal blockade. A multicenter phase I study [44] demonstrated antitumor activity in HER2-positive solid tumors, including BTC. Among 22 BTC patients (99% with IHC 3+ or IHC 2+/FISH+), the objective response rate (ORR) was 38%, with an mPFS of 3.5 months. Grade ≥ 3 treatment-related adverse events occurred in only 1% of patients, primarily mild diarrhea and infusion reactions, indicating a favorable safety profile. Another non-randomized multicenter phase IIa study [45] evaluated pertuzumab plus trastuzumab in 39 previously treated HER2-positive metastatic BTC patients, reporting an ORR of 23%, mPFS of 4.0 months, and mOS of 10.9 months. However, grade ≥3 adverse events were observed in 46% of patients. Subgroup analysis suggested better efficacy in gallbladder and ampullary cancers, potentially correlated with higher HER2 copy numbers.

Additionally, anti-angiogenic therapy has gained interest in BTC research. Surufatinib is an oral small-molecule inhibitor targeting VEGFR1/2/3, FGFR1, and CSF1R, exerting both anti-angiogenic and immunomodulatory effects. A multicenter single-arm phase II study conducted in China evaluated surufatinib as a second-line therapy in 39 patients with unresectable or metastatic BTC who had failed first-line chemotherapy [46]. The 16-week progression-free survival rate was 46.33%, but grade ≥3 treatment-related adverse events occurred in 69.2% of patients, including hypertension and proteinuria, which were mostly manageable with supportive care and dose adjustments. Subgroup analysis indicated that patients with baseline CA19-9 ≤1000 IU/mL and those with intrahepatic cholangiocarcinoma may derive greater benefit (mPFS: 5.1 months vs. 3.3 months), suggesting that this population might be more suitable for such targeted therapy.

### 6.3. Immunotherapy

In a phase II two-arm clinical trial [47], investigators evaluated the efficacy and safety of durvalumab (an anti-PD-L1 antibody) and tremelimumab (an anti-CTLA-4 antibody), with or without paclitaxel, in patients with advanced biliary tract cancer (BTC) who had progressed on platinum-based chemotherapy. Tremelimumab enhances T-cell activation and antitumor immune responses by blocking CTLA-4 signaling and belongs to the same class of immune checkpoint inhibitors as nivolumab. The study enrolled 20 patients randomized to either a triple-therapy group (durvalumab + tremelimumab + paclitaxel) or a dual immunotherapy group (durvalumab + tremelimumab), with 10 patients in each arm. The cohort consisted predominantly of intrahepatic cholangiocarcinoma (70%), and most patients had previously received gemcitabine plus cisplatin (65%) or gemcitabine plus oxaliplatin (25%). However, grade ≥3 treatment-related adverse events occurred in 60% of patients in the triple-therapy group, and 50% discontinued treatment due to toxicity, indicating an unfavorable safety profile that led to premature study termination. Thus, the combination of paclitaxel with dual immune checkpoint inhibitors requires further optimization, including dose adjustment and improved toxicity management.

In contrast, nivolumab monotherapy has demonstrated modest antitumor activity in advanced refractory BTC. A multicenter phase II study [48] enrolled 54 patients who had received 1–3 prior lines of systemic therapy, 59% of whom had intrahepatic cholangiocarcinoma. Treatment consisted of nivolumab 240 mg every two weeks for the first 16 weeks, followed by maintenance therapy with 480 mg every four weeks. The results showed an objective response rate (ORR) of 22%, a median progression-free survival (mPFS) of 3.68 months, and a median overall survival (mOS) of 14.24 months. Subgroup analysis indicated that patients with PD-L1 expression ≥1% had significantly prolonged progression-free survival (HR = 0.23, *p* < 0.001). Grade ≥3 treatment-related adverse events occurred in 17% of patients, suggesting a manageable safety profile that supports its use in later-line therapy.

To further enhance efficacy, dual immune checkpoint inhibition has attracted considerable interest. A multicenter, non-randomized phase II trial evaluated nivolumab in combination with ipilimumab (the first clinically approved CTLA-4 inhibitor, often used together with PD-1 inhibitors) in 39 patients with advanced BTC, 33 of whom had received at least one prior line of therapy [49]. The regimen consisted of nivolumab 3 mg/kg plus ipilimumab 1 mg/kg every three weeks for four doses, followed by maintenance nivolumab 3 mg/kg every two weeks. The results demonstrated an ORR of 23%, mPFS of 2.9 months, and mOS of 5.7 months. Responses were observed only in patients with intrahepatic cholangiocarcinoma or gallbladder cancer, with no responses in those with extrahepatic cholangiocarcinoma. Grade ≥3 treatment-related adverse events occurred in 15% of patients, indicating an acceptable safety profile and potential antitumor activity in specific anatomical subtypes.

Another phase II study [50] focusing on gallbladder cancer (SWOG 1609 Cohort 48) further validated this combination. The trial included 19 patients with previously treated advanced gallbladder cancer who received nivolumab (240 mg every two weeks) plus ipilimumab (1 mg/kg every six weeks). The results showed an ORR of 16%, mOS of 7.0 months, and a 6-month progression-free survival rate of 26%. Treatment-related adverse events occurred in 84% of patients, with fatigue and anemia being most common; among grade ≥3 events, elevated AST was predominant. Patel SP and colleagues concluded that the combination was well-tolerated and exhibited promising clinical activity in refractory advanced gallbladder cancer.

In summary, although immune monotherapy or dual immune combination therapy has demonstrated modest efficacy and a manageable safety profile in advanced BTC, treatment responses vary significantly across anatomical subtypes. Patients with intrahepatic cholangiocarcinoma and gallbladder cancer derive more substantial benefit, while those with extrahepatic cholangiocarcinoma show limited responses. Moreover, triple chemoimmunotherapy regimens face considerable challenges due to safety concerns. Further prospective studies are needed to optimize combination strategies, improve toxicity management, and identify predictive biomarkers to enable personalized treatment and improve patient outcomes.

### 6.4. Combination of Targeted Therapy and Immunotherapy

In a Chinese multicenter randomized phase II clinical trial [51], investigators evaluated the efficacy and safety of sintilimab (a PD-1 inhibitor that enhances T cell-mediated antitumor immunity by blocking the PD-1/PD-L1 pathway) combined with anlotinib (a multi-target tyrosine kinase inhibitor targeting VEGFR, FGFR, PDGFR, and other pathways, with antiangiogenic and antiproliferative effects) and gemcitabine/cisplatin (SAGC regimen) versus gemcitabine/cisplatin alone (GC regimen) as first-line treatment for unresectable or metastatic biliary tract cancer. The study enrolled 80 patients, randomized 1:1. The GC group received gemcitabine 1000 mg/m^2^ (days 1 and 8) plus cisplatin 25 mg/m^2^, repeated every 3 weeks; the SAGC group received GC plus sintilimab 200 mg every 3 weeks and oral anlotinib (initial dose 10 mg/day, later adjusted to 8 mg/day in a 2-weeks-on/1-week-off schedule). Treatment was administered for 8 cycles.

Results showed that the SAGC regimen significantly prolonged median progression-free survival (mPFS: 8.5 months vs. 6.3 months; HR = 0.48, 95% CI: 0.22–0.64, *p* = 0.005) and improved the objective response rate (ORR: 51.4% vs. 29.4%, *p* = 0.033) compared to GC, with a longer median duration of response (9.1 months vs. 3.4 months). Notably, after the anlotinib dose was reduced from 10 mg to 8 mg, the ORR further increased (54.5% vs. 38.8%), median overall survival showed a trend toward improvement (14.9 months vs. 9.3 months, HR = 0.49, *p* = 0.055), and safety significantly improved: the incidence of grade ≥3 thrombocytopenia decreased from 38.9% to 13.6%, and grade 4 adverse events decreased from 23.5% to 13.0%. Preclinical studies suggested that lower-dose anlotinib may enhance synergistic effects with immunotherapy by avoiding excessive vascular pruning, improving immune cell infiltration in the tumor microenvironment, and modulating immunosuppressive factors. This study not only explores a novel first-line strategy for advanced BTC but also highlights the potential of combining antiangiogenic agents with immune checkpoint inhibitors, though these findings require further validation in large-scale phase III randomized controlled trials.

In contrast, another study [52] focused on the efficacy and safety of the bifunctional fusion protein SHR-1701 (simultaneously targeting PD-L1 and TGF-β) combined with the multi-target TKI famitinib in pretreated patients with advanced biliary tract cancer (BTC) and pancreatic ductal adenocarcinoma (PDAC). This single-arm phase II trial enrolled 51 patients (27 BTC and 24 PDAC), all receiving oral famitinib 20 mg daily plus intravenous SHR-1701 every 3 weeks. In the BTC cohort, 81.5% had liver metastases, and most patients had received 1–2 prior lines of therapy. Results showed an ORR of 28% and a disease control rate (DCR) of 80% in BTC patients, with mPFS of 5.1 months (95% CI: 2.6–7.6) and mOS of 16.0 months. The overall incidence of grade ≥3 treatment-related adverse events was 29.4%, lower than that reported in similar studies (e.g., the LEAP-005 trial). Yi L et al. concluded that this combination had a manageable safety profile and employed an inflammation/immune score (I/M score) for efficacy prediction, demonstrating potential for further clinical development (Table 4).

### 6.5. Novel Therapeutic Approaches

Aspirin may delay the progression and metastasis of biliary tract cancer (BTC) by inhibiting cyclooxygenase-2 (COX-2) activity and platelet aggregation, thereby interfering with tumor-associated inflammation and microthrombus formation. A retrospective analysis [53] based on data from the UK Clinical Practice Research Datalink (CPRD) from 1990 to 2017, involving adult BTC patients, demonstrated that aspirin use from the time of diagnosis was significantly associated with reduced all-cause mortality (gallbladder cancer: HR = 0.63; cholangiocarcinoma: HR = 0.71; ampullary cancer: HR = 0.44; mixed tumors: HR = 0.68). Notably, survival benefits were more pronounced in new users compared to long-term users. The study included 2934 patients, comprising 667 gallbladder cancers (23%), 1559 cholangiocarcinomas (53%), 224 ampullary cancers (8%), and 484 mixed tumors (16%), providing a relatively large sample size and robust statistical power. However, key covariates such as cancer stage, systemic treatment regimens, and performance status were not adjusted for, leaving potential residual confounding. Thus, these findings require validation through prospective randomized controlled trials.

In the realm of local therapy, proton beam therapy (PBT) leverages the physical properties of the Bragg peak to precisely target tumors while maximally sparing surrounding normal liver and gastrointestinal tissues, making it particularly suitable for anatomically complex extrahepatic cholangiocarcinoma (ECC). A recent Japanese study [54] reported that among 201 patients with unresectable ECC treated with a median dose of 67.5 Gy in 25 fractions, the median overall survival (mOS) reached 20.0 months, with a 2-year progression-free survival (PFS) rate of 23.0%. Notably, patients aged >75 years achieved a 2-year overall survival rate of 41.3%, comparable to younger patients, suggesting that PBT is equally safe and effective in the elderly population. The incidence of acute grade 3 adverse events was only 4.0%, significantly lower than with conventional radiotherapy. Multivariate analysis identified tumor diameter <3 cm, Child-Pugh A liver function, and a distance >2 cm between the tumor and gastrointestinal tract as significant predictors of improved survival, offering a new radical treatment option for unresectable ECC, especially in elderly patients or those with comorbidities.

Recent research on the tumor microbiome has provided new perspectives on the mechanisms and treatment strategies for BTC. Okuda S. et al. [55] analyzed 15 patients who underwent radical resection for pancreatic or biliary tract cancer and found that the microbial composition was highly similar between tumor tissues and adjacent normal tissues, while fluids such as bile and pancreatic juice contained an abundance of uncultured or unidentified bacteria. Notably, Akkermansia was detected exclusively in the bile of BTC patients and was significantly associated with external biliary drainage (75% in the drainage group vs. 14.3% in the non-drainage group, *p* = 0.041), suggesting that specific microbial taxa may serve as prognostic markers or therapeutic targets. Another study [56] revealed that the bile microbial community structure and functional pathways (e.g., peptidoglycan and sphingolipid metabolism) in cholangiocarcinoma and pancreatic cancer patients were significantly distinct from those in patients with common bile duct stones, indicating that microbes may participate in BTC pathogenesis through metabolic regulation. Further reviews propose that investigating microbial metabolites and the gut-liver axis interaction mechanisms may elucidate the pathogenesis of BTC and lead to novel intervention strategies, such as microbiota modulation combined with immunotherapy [57].

## 7. Conclusions

The management of advanced biliary tract cancer (BTC) has witnessed considerable advances in recent years, particularly with the introduction of targeted therapy, immunotherapy, and combination strategies on the basis of traditional chemotherapy, providing patients with more individualized treatment options. However, there remains no unified standard for third-line therapy, and multiple strategies each present distinct advantages and limitations.

Currently, biomarker-directed targeted therapies (e.g., targeting FGFR2, IDH1, BRAF V600E, and HER2 alterations) and immunotherapy (especially PD-1/PD-L1 inhibitors) have emerged as pivotal forces reshaping the clinical landscape of advanced BTC, delivering significant survival benefits in specific patient subsets. Meanwhile, various combination approaches—such as immunotherapy plus chemotherapy, antiangiogenic agents combined with immunotherapy, dual immune checkpoint inhibition, and novel bifunctional agents targeting emerging pathways like TGF-β—have demonstrated potential beyond conventional chemotherapy in clinical trials, signaling a paradigm shift in therapeutic concepts.

Nevertheless, multiple challenges persist. First, the populations that benefit from targeted and immunotherapeutic agents remain relatively limited, with efficacy varying considerably across anatomical subtypes and molecular profiles. Second, many promising treatments are currently supported predominantly by Phase II evidence, underscoring the urgent need for validation through large-scale Phase III randomized controlled trials. Additionally, treatment-related toxicities, mechanisms of resistance, and disparities in diagnostic and therapeutic access and equity worldwide represent critical barriers that must be overcome to achieve personalized precision medicine.

Future research should focus on the following directions: first, conducting large-scale randomized controlled trials to identify optimal populations and combination strategies; second, elucidating mechanisms of resistance to develop next-generation inhibitors or rational combination regimens; third, promoting the clinical integration of multi-omics technologies and liquid biopsies to enable dynamic and precise monitoring of treatment response and prognosis; fourth, strengthening translational research to explore novel therapeutic targets such as the microbiome and metabolic reprogramming; and fifth, emphasizing real-world evidence and international collaboration to facilitate guideline updates and homogenization of global practice standards.

In conclusion, the treatment of advanced BTC is progressively transitioning from a “one-size-fits-all” chemotherapy-based approach toward a new era characterized by individualized and multifaceted strategies. Through continued optimization of treatment regimens, refinement of biomarker selection, and improved management of treatment-related adverse events, there is growing potential to achieve meaningful survival benefits for patients with advanced BTC.

## Figures and Tables

**Table 1:** First-line treatment regimen.

Regimen	Applicable Population	Design	mOS	Hazard Ratio *p* Value	mPFS	Hazard Ratio *p* Value	ORR	*p* Value	Source
Durvalumab + GEM + DDP	U/M	Durvalumab + GEM + DDP GEM + DDP	12.9 m 11.3 m	HR = 0.80 *p* = 0.021	7.2 m 5.7 m	HR = 0.75 *p* = 0.001	26.7% 18.7%	*p* = 0.007	TOPAZ-1
Pembrolizumab + GEM + DDP	U/M	Pembrolizumab + GEM + DDP GEM + DDP	12.7 m 10.9 m	HR = 0.83 *p* = 0.0034	6.3 m 5.8 m	HR = 0.86 *p* = 0.0038	29.0% 24.0%	*p* = 0.0029	KEYNOTE-966
GEM + DDP	NI	GEM + DDP GEM	11.7 m 8.1 m	HR = 0.64 *p* < 0.001	8.0 m 5.0 m	HR = 0.63 *p* < 0.001	26.1% 15.5%	*p* = 0.006	ABC-02
Targeted Therapy	Patients with alterations *	Refer to Table 2							

DDP: Cisplatin; GEM: Gemcitabine; U/M: Unresectable or metastatic BTC; mOS: Median Overall Survival; mPFS: Median Progression Free Survival; ORR: Overall Response Rate; HR: Hazard ratio; m: Months; NI: Immune-combination regimens are not applicable; *: Such as MSI-H/dMMR, NTRK fusions, FGFR2 alterations, etc.

**Table 2:** Targeted therapy strategies for biliary tract cancer.

Biomarker	Regimen	Study Population	Treatment Group	mDoR	mPFS	mOS	ORR	DCR	Trial
MSI-H/dMMR	Pembrolizumab *	Pre-treated Patients	SA	N	4.1 m	23.5 m	39.60%	-	KEYNOTE-158
NTRK Gene Fusion	Larotrectinib *			49.3 m	35.4 m	N	75.00%	-	LOXO-TRK-01,NAVIGATE
	Entrectinib *			20.0 m	13.8 m	33.8 m	63.00%	-	STARTRK-1/2,ALKA-372-001
FGFR2 Fusion/ Rearrangement	Pemigatinib *			7.5 m	6.9 m	21.1 m	35.50%	82.00%	FIGHT-202
	Futibatinib *			9.5 m	8.9 m	20.0 m	41.70%	82.50%	FOENIX-CCA2
	Infigratinib			5.0 m	7.3 m	12.2 m	23.10%	84.30%	CBGJ398X2204
IDH1 Mutation	Ivosidenib *		Ivosidenib PB	-	2.7 m 1.4 m (HR = 0.37, *p* < 0.0001)	10.3 m 7.5 m (HR = 0.49, *p* < 0.0001)	2.4% *p* = 0.283	53.2% *p* < 0.0001	ClarlDHy
BRAF V600E Mutation	Dabrafenib * + Trametinib *		SA	8.7 m	8.9 m	13.5 m	51%	79%	ROAR

N: Not Reached; SA: Single-Arm Cohort; “-”: Not reported; mDoR: Median Duration of Response; mOS: Median Overall Survival; mPFS: Median Progression Free Survival; ORR: Overall Response Rate; m: Months; PB: Placebo; *: Recommended by the ESMO Guidelines.

**Table 3:** Second-line treatment regimens.

Treatment Population	Treatment Strategy	Regimen	mOS	Hazard Ratio *p* Value	mPFS	Hazard Ratio *p* Value	ORR	Trail
With actionable targets	Refer to Table 2							
Without actionable targets	FOLFOX	FOLFOX +ASC ASC	6.2 m 5.3 m	HR = 0.69 *p* = 0.031	4.0 m 2.0 m	HR = 0.63 *p* = 0.006	5% 0	ABC-06
	FOLFIRI	Irinotecan +Leucovorin +5-FU	10.6 m	-	4.6 m	-	15%	Phase II single-arm
	Nab-Paclitaxel +Gemcitabine	Nab-Paclitaxel +Gemcitabine	7-9 m	-	3-5 m	-	10-20%	Retrospective analysis
	Capecitabine	Capecitabine	5.3 m	-	2.3 m	-	5.60%	Retrospective analysis
	S-1	S-1	7.6 m	-	2.7 m	-	10%	Phase II single-arm

ASC: Active Symptom Control; mOS: Median Overall Survival; mPFS: Median Progression Free Survival; ORR: Overall Response Rate; HR: Hazard.

**Table 4:** Clinical trials in the refractory setting in patients with biliary tract cancer.

Clinical Trail	N	Regimen	mOS	Hazard Ratio *p* Value	mPFS	Hazard Ratio *p* Value	ORR	*p* Value
chemotherapy								
NIFTY Trial Jaewon Hyung, Ilhwan Kim et al. *JAMA Oncol.* 2023 [40]	174	nal-IRI + FU/LV PB	8.6 m 5.3 m	HR = 0.68 *p* = 0.02	3.9 m 1.6 m	HR: 0.51 *p* < 0.001	19.3% 2.3%	*p* < 0.001
targeted therapy								
FIGHT-202 Trial Abou-Alfa GK et al. *Lancet Oncol.* 2020 [29]	146	pemigatinib	21.1 m	-	6.9 m	-	35.50%	-
FOENIX-CCA2 Trial Goyal L et al. *N. Engl. J. Med.* 2023 [30]	103	futibatinib	20.0 m	-	8.9 m	-	41.70%	-
ClarIDHy Goyal L et al. *N. Engl. J. Med.* 2023 [30]	185	Ivosidenib PB	10.3 m 7.5 m	HR = 0.49 *p* < 0.0001	2.7 m 1.4 m	HR: 0.37 *p* < 0.0001	2.4% 0	-
ROAR Trial Vivek Subbiah et al. *Lancet Oncol.* 2020 [42]	43	Dabrafenib+ trametinib	13.5 m	-	8.9 m	-	51.00%	-
Zanidatamab-related Funda Meric-Bernstam et al. *Lancet Oncol.* 2022 [44]	86	zanidatamab	-	-	5.4 m	-	37.00%	-
Surufatinib-related Jianming Xu et al. *Cancer* 2021 [46]	39	surufatinib	6.9 m	-	3.7 m	-	0.00%	-
immunotherapy								
Nivolumab-related Richard D. Kim et al. *JAMA Oncol.* 2020 [48]	54	nivolumab	14.24 m	-	3.68 m	-	22.00%	-
SWOG 1609 cohort 48 Sandip P. Patel et al. *Cancer* 2024 [50]	19	Nivolumab+ ipilimumab	7 m	-	1.8 m	-	16.00%	-
immunotherapy combined with chemotherapy								
IMMUNOBIL PRODIGE 57 Trial Alice Boilève et al. *Eur. J. Cancer* 2021 [47]	20	Durvalumab + tremelimumab + paclitaxel PB	-	-	-	-	10.00%	-
immunotherapy combined with targeted therapy								
SAGC Trial Jaewon Hyung, Ilhwan Kim et al. *JAMA Oncol.* 2023 [40]	80	Sintilimab + anlotinib + GC GC	13.2 m 13.7 m	HR: 1.04 *p* = 0.895	8.5 m 6.3 m	HR: 0.48 *p* = 0.005	51.4% 29.4%	*p* = 0.033
SHR-1701 combined with famitinib Lixia Yi et al. *Signal Transduct. Target. Ther.* 2024 [52]	27	SHR-1701+ famitinib	16.0 m	-	5.1 m	-	28.00%	-

“-”: Not reported; mOS: Median Overall Survival; mPFS: Median Progression Free Survival; ORR: Overall Response Rate; HR: Hazard.
